# HER2 status in advanced gastric carcinoma: A retrospective multicentric analysis from Sicily

**DOI:** 10.3892/ol.2013.1611

**Published:** 2013-10-10

**Authors:** A. IENI, V. BARRESI, G. GIUFFRÈ, R.A. CARUSO, S. LANZAFAME, L. VILLARI, E. SALOMONE, E. ROZ, D. CABIBI, V. FRANCO, G. CERTO, A. LABATE, C. NAGAR, E. MAGLIOLO, B. BROGGI, C. FAZZARI, F. ITALIA, G. TUCCARI

**Affiliations:** 1 Department of Human Pathology, University of Messina, Messina, Italy; 2 Pathological Anatomy Unit, ASP 5 Messina, Messina, Italy; 3 Department of Anatomy, Diagnostic Pathology, Legal Medicine and Public Health, University of Catania, Catania, Italy; 4 Pathological Anatomy Unit, Vittorio Emanuele II University Hospital, Catania, Italy; 5 Health Home Service ‘La Maddalena’, University of Palermo, Palermo, Italy; 6 Department of Human Pathology, University of Palermo, Palermo, Italy; 7 Health Home Service ‘IOMI-Cappellani’, Messina, Italy; 8 Pathological Anatomy Unit, ASP 6 Palermo, Palermo, Italy; 9 Pathological Anatomy Unit, ASP 8 Siracusa, Siracusa, Italy; 10 Humanitas Center of Oncology, Catania, Italy; 11 Oncopathology Laboratory, Floridia, Siracusa, Italy; 12 Cytodiagnostics and Molecular Biology Interdepartmental programme, ‘Polyclinic G Martino’ University Hospital, Messina, Sicily, Italy

**Keywords:** HER2 status, gastric adenocarcinoma, trastuzumab, Sicily

## Abstract

According to the ToGA trial, HER2 has been shown to be predictive for the success of treatment with trastuzumab in advanced gastric cancer (AGC). A number of studies have analyzed HER-2/neu overexpression in gastric carcinoma and identified the rate of HER2 positivity to be markedly varied. To date, the prevalence of HER2 overexpression in Sicilian people with AGC is unknown. Therefore, in the present study, a retrospective immunohistochemical analysis of HER2 was performed in a cohort of 304 AGC samples that were obtained from the archives of 10 Sicilian anatomopathological diagnostic units in order to verify the positive rate of HER2-positive cases. Furthermore, the characteristics of histotype, grade, stage and Ki-67 expression were also analyzed. HER2 overexpression was encountered in 17.43% of all the gastric adenocarcinomas, which was consistent with the results that have been reported elsewhere in the literature. A progressive increase in HER2 overexpression was observed, from the poorly cohesive histotype to the tubular adenocarcinomas and gastric hepatoid adenocarcinomas. HER2 overexpression was significantly associated with a high grade, advanced stage and high Ki-67 labeling index. Further investigations performed jointly by pathologists and oncologists within the geographical area of the present study should confirm that the association of trastuzumab with chemotherapy results in an improvement of survival in patients with AGC.

## Introduction

HER-2 gene amplification and protein overexpression have been indicated as the targets for therapy with the anti-HER2 humanized monoclonal antibody, trastuzumab, in various cancers (1–9). In these studies, a wide range of HER-2 expression has been described with controversial data (9). However, in an open-label international phase 3 randomized controlled trial that was undertaken in 122 centers within 24 countries, patients with advanced gastric or gastroesophageal junction carcinomas were studied in order to verify whether the tumors demonstrated an overexpression of HER-2 protein, as detected by immunohistochemistry or gene amplification using fluorescence *in situ* hybridization (FISH) (10). In particular, the randomized ToGA study revealed a 26% reduction in the risk of mortality when trastuzumab was added to the chemotherapy regime (hazard ratio, 0.74) for treating advanced gastric carcinomas (AGCs) (10). Although the reported rates of HER-2 overexpression appear to be variable, there is a general agreement with regard to a higher HER-2 positivity in gastroesophageal junction cancer (24–35%) compared with gastric carcinoma (9.5–21%) (11–13). Furthermore, common gastric tumors that are classified as intestinal types are more likely to be HER-2 positive (16–34%) compared with diffuse (2–7%) or mixed (5–20%) types (11–12,14,15), although certain aggressive variants, including hepatoid gastric carcinomas, have been shown to exhibit the highest HER-2 immunoreactivity (16).

To date, no data with regard to the HER2 status in AGCs are available from the geographical area of a Mediterranean region, such as Sicily, though a mean decrease of 29.7% has been registered in the comparison between 1993–1995 and 2003–2005, independently from the gender of patients (17). Therefore, the present study analyzed the HER2 status in a cohort of 304 surgical cases of advanced/metastatic gastric carcinomas that were obtained from the archives of 10 Sicilian anatomopathological units, in order to verify the positive rate of HER2-positive cases, taking into consideration the characteristics of the histotype, grade, stage and Ki-67 expression.

## Materials and methods

### Samples

A total of 304 surgically-treated patients with gastric adenocarcinoma, who were not administered neoadjuvant chemotherapy, were selected from the files of 10 Sicilian anatomopathological diagnostic units. The patients who succumbed within 30 days of surgery (post-operative mortality) were excluded from the study. Informed consent was obtained from all the patients that were studied. Furthermore, the study was purely observational and no medical interventions were performed. Approval for the study was obtained from the Local Ethics Committees of the University of Messina (Messina, Italy), University of Catania (Catania, Italy) and University of Palermo (Palermo, Italy).

The tumors were taken from an equal number of patients, 183 of which were male and 121 of which were female, with a mean age of 68.3 years (range, 41–93 years). The tumor was localized in the antrum of the stomach in 151 patients, the body in 136 and the fundus in 17, 11 of which were located at the gastroesophageal junction. All the gastric surgical specimens were fixed in 10% neutral formalin for 24–48 h and paraffin embedded at 56°C. The histotypes, according to the WHO classification (18), grade and staging were also available. A total of 183 tubular/papillary/mucinous adenocarcinomas (PTM adenocarcinomas), 98 poorly cohesive carcinomas and 23 cases of rare variants of adenocarcinomas, including 14 hepatoid adenocarcinomas (HACs) and nine mitochondrion-rich adenocarcinomas (MRCs), were analyzed. HACs are identified as adenocarcinomas that exhibit only a solid pattern of large polygonal eosinophilic cells, without foci of tubular/papillary differentiated areas and a clear cytoplasm within the cells. This hepatoid variant was validated by an immunohistochemical (IHC) analysis for α-1-fetoprotein and HepPar1 antisera. The MRCs were morphologically characterized by ultrastructural observations, including junctional complexes and desmosomes with numerous mitochondria in the cytoplasm of the tumor cells, which were mainly concentrated in the supranuclear region. This oncocytic variant was validated by an immunohistochemical analysis for anti-mitochondrial antibody.

### HER2 determination

HER-2 status was evaluated by IHC immunostaining on 3-μm thick sections that were mounted on silane-coated slides using HercepTest™ (Dako, Glostrup, Denmark). An antigen retrieval pre-treatment was performed by three changes in 0.01 M citrate buffer (pH 6.0) in a microwave oven at 750 W. Each immunostained section was evaluated by the following score: 0, absent staining, 1+, faint and discontinuous membranous staining in <10% of neoplastic elements; 2+, light to moderate lateral, basolateral or complete membranous staining in >10% of neoplastic elements; and 3+, strong, intense lateral, basolateral or complete staining in >10% of neoplastic elements. For reproducible intensity scoring, it is advised to apply the magnification rule algorithm, which has been previously described (15). All the cases that were considered equivocal (2+) were further assessed using the FISH test (pharmDx; Dako) in three reference laboratories. Gene amplification was recorded when the HER2:CEP17 signal ratio was >2.0.

### Additional immunohistochemistry

The HAC samples were tested using polyclonal rabbit anti-human α-1-fetoprotein (clone A000802-29) and monoclonal mouse anti-human hepatocyte (Clone OCH1E5) (Dako). A strongly positive immunostaining pattern that was diffuse or focally granular was encountered in the cytoplasm of the neoplastic cells. The MRC samples exhibited a positive immunostaining pattern that was localized in the cellular supranuclear region by mouse polyclonal anti-human mitochondria antibody (clone MTCO2).

In parallel sections that were obtained from the same tissue blocks, Ki-67 antigen was unmasked by the previously cited retrieval procedure. Ki-67 antiserum (clone MIB-1; w.d. 1:50; Dako) was applied for 30 min at room temperature. The Ki-67 labeling index (LI) was calculated as the mean percentage by counting the stained nuclei of 1,000 tumor cells in three representative neoplastic fields. All degrees of nuclear staining intensity were taken into consideration. The median Ki-67 LI value (30%) was utilized as the cut-off point to define low and high Ki-67 expression.

### Statistical analysis

Statistical analysis was performed by χ^2^ test to analyze the associations between HER2 status and the clinicopathological parameters. P<0.05 was considered to indicate a statistically significant difference. The data were analyzed using the SPSS package, version 6.1.3 (SPSS, Inc., Chicago, IL, USA).

## Results

**Figure 1:**
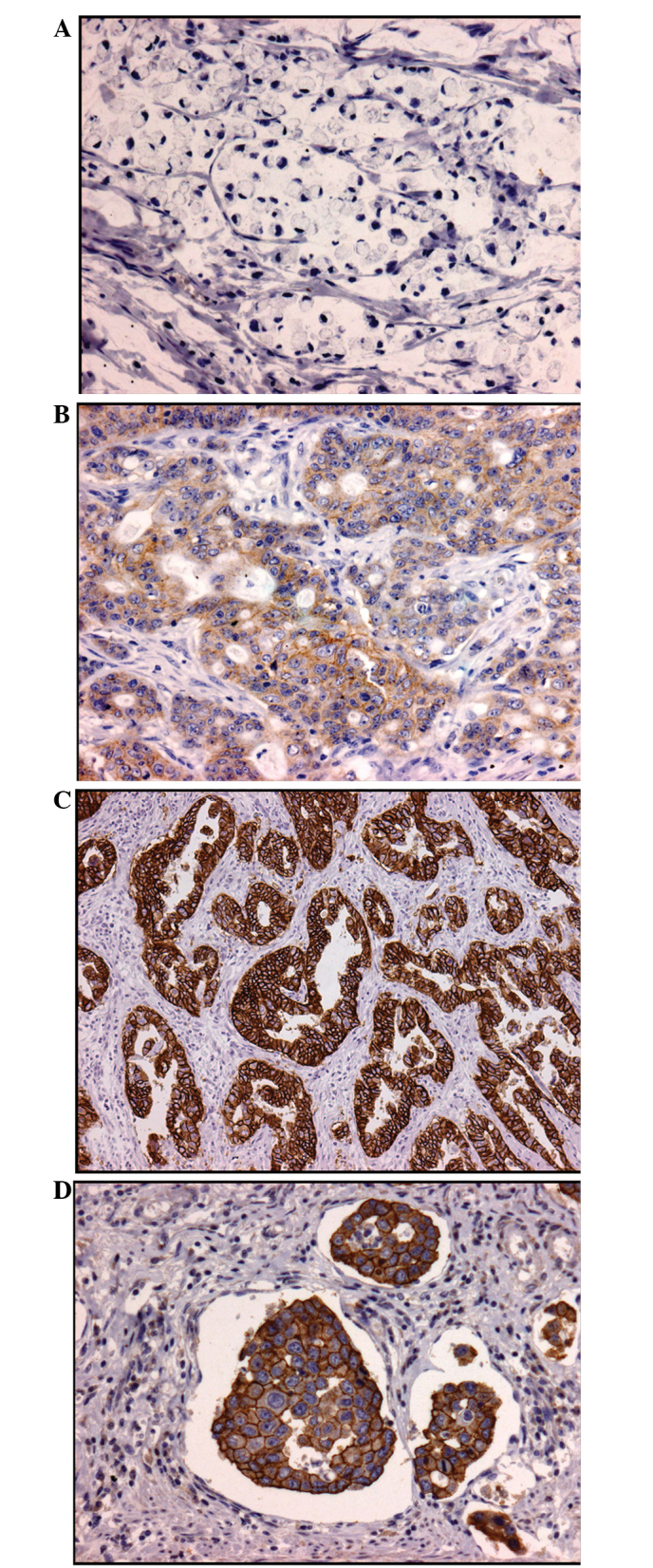
(A) Neoplastic elements of the poorly cohesive histotype showing HER2 negativity (x400). (B) Light-moderate incomplete HER2 2+ staining was appreciated in the tubular areas of the adenocarcinomas (x400). (C) Strong intense complete and diffuse HER2 staining was encountered in AGC at a lower magnification (x150). (D) Neoplastic thrombi showing 3+ HER2 overexpression (x400). AGC, advanced gastric carcinoma.

Taking into consideration the HER2-positive rate, a range of variability of 7.69–21.7% was identified in the various anatomopathological units, with a mean value of 17.43%. HER2 overexpression was encountered in 53 of the total AGC cases, independently from the histotype. In detail, a progressive increase in the oncoprotein immunoreactivity was observed, from the frequently HER2-negative poorly cohesive histotype (3.5%; Fig. 1A) to MRCs (11.1%), tubular/papillary adenocarcinomas (31.3%; Fig. 1B and C) and HACs (42.9%). HER2 immunoreactivity was also evident in the angiolymphatic neoplastic thrombi (Fig. 1D). Finally, HER2 overexpression was significantly associated with a high grade (P=0.011), advanced stage (P=0.002) and high Ki-67 LI value (P=0.015).

## Discussion

Numerous studies have documented HER2 amplification in AGC, although the association with the survival or TNM status of patients remains debatable (15,19–24). Giuffrè *et al* previously encountered a HER2 overexpression rate of 21.10% in a smaller cohort of gastric adenocarcinomas that were obtained from a single pathological unit (16), which is consistent with the results that have been reported elsewhere in the literature (11,19,21,25–28), in which the mean HER2 positivity rate, using FISH or chromogenic *in situ* hybridization, was 19.2% (range, 7.1–42.6%) (25). In the present study, which analyzed a larger sample of AGC from a number of varying institutions within the same geographical area, HER2 overexpression was identified in 53 (17.43%) of the 304 samples of AGC. This latter rate was marginally lower than the mean HER2 positivity that has been previously cited (25). However, taking into consideration only the IHC studies that are available in the literature, which have been performed on 3264 AGC samples (25), the mean HER2-positive rate was 17.6% (range, 6.8–34.0%), which is equivalent to the result of the present study. Consequently, a non-significant variation in HER2 determination should be attributed to the subjective interpretation, as well as the scoring of the immunohistochemical results, of the AGC samples that were observed in the geographical area of the present study. The achieved reproducibility between the various laboratories within the present study was predominantly due to standardized fixation methods and times for the tissue samples. Furthermore, the application of the magnification rule (15) in the IHC observations and the determination of HER2 gene amplification by FISH, which was performed exclusively in three well-trained anatomopathological institutions, have further contributed to reduce the inter-observer variability and to guarantee an accurate evaluation of the HER2 status. However, the requirement to improve HER2 expression and gene amplification in gastric cancer has been also been indicated by a consortium of expert pathologists in other European countries (15,25,29), which is similar to the retrospective purpose of the present study.

In the present study, a progressive increase in HER2 immunoreactivity was observed, from the poorly cohesive histotype (3.5%) to MRCs (11.1%), tubular/papillary adenocarcinomas (31.3%) and HACs (42.9%). Furthermore, HER2 overexpression was also significantly associated with a high grade, stage and Ki-67 LI value. Thus, the HER2 status may represent an additional morphological parameter that is able to affect the mortality of patients with gastric cancer. Although there is a possibility that HER2 overexpression in AGC may emerge as an independent prognostic parameter, this requires clinical oncological outcomes that are currently unavailable for the present casuistry. However, in a previous multivariate analysis, HER2 overexpression was identified as an independent unfavorable prognostic variable for adenocarcinomas as a whole and also for the unusual hepatoid variant (16).

In conclusion, further investigations that are jointly performed by pathologists and oncologists present within the geographical area of the current study should confirm that the association between trastuzumab and chemotherapy determines an improvement in survival for patients with AGC showing amplification or overexpression of the HER2 protein.
